# Supplementary material for: Dynamic inference of cell developmental complex energy landscape from time series single-cell transcriptomic data
Source: PLoS Comput Biol. 2022 Jan 24;18(1):e1009821. doi: 10.1371/journal.pcbi.1009821 (PMC8812873; doi:10.1371/journal.pcbi.1009821)
Supplement: S1 Text — This document provides detailed description of the parameter estimation and pseudocode for the GrapFP algorithm. (PDF) [file pcbi.1009821.s001.pdf]

Dynamic inference of cell developmental complex energy landscape from time series single-cell transcriptomic data

Qi Jiang, Shuo Zhang, Lin Wan

Supporting Information
S1 Text. Details for the parameter estimation of GraphFP

I: An adjoint method based gradient algorithm for the parameter estimation

GraphFP formulate the estimation problem as follows

$$\boldsymbol{\theta}^* = \arg\min_{\boldsymbol{\theta}} \int_{t_1}^{t_f} \frac{1}{2} \sum_{\{i,j\}\in E} \left(F_i(\boldsymbol{p}(t)) - F_j(\boldsymbol{p}(t))\right)^2 \cdot g_{ij}\left(\boldsymbol{p}\left(t\right)\right) dt + \sum_{l=2}^{f} \lambda_l \mathrm{KL}\left(\boldsymbol{p}\left(t_l\right) \parallel \boldsymbol{p}^l\right), \quad (1)$$

subject to the constraints

$$\frac{d\boldsymbol{p}(t)}{dt} = \left(\sum_{j\in N(i)} \left(F_j(\boldsymbol{p}(t)) - F_i(\boldsymbol{p}(t))\right) g_{ij}(\boldsymbol{p}(t)) \right)_{i=1}^{n}, \quad (2)$$

$$\boldsymbol{p}(t_1) = \boldsymbol{p}^1, \quad (3)$$

We first write the above formulation in the vector and matrix forms to make it concise. Note that by defined mathematical form of $g_{ij}(\boldsymbol{p})$, Equation (2) can also be expressed as

$$\frac{dp_i(t)}{dt} = \sum_{j\in N(i)} \left[\frac{p_j\left(t\right) + p_i\left(t\right)}{2} \cdot \left(F_j(\boldsymbol{p}(t)) - F_i(\boldsymbol{p}(t))\right) + \frac{p_j\left(t\right) - p_i\left(t\right)}{2} \cdot \left|F_j\left(\boldsymbol{p}\left(t\right)\right) - F_i\left(\boldsymbol{p}\left(t\right)\right)\right| \right].$$

We define the incidence matrix of cell state-transition graph G, an undirected graph, as $\boldsymbol{A} \in \{0,1\}^{n\times|E|}$:

$$A_{ke} = \begin{cases} 1 & \exists l, \quad \text{s.t. } e = (k,l) \in G; \\ 0 & \text{otherwise.} \end{cases}$$

We also introduce a directed graph $\hat{G}$ with its incidence matrix $\boldsymbol{B} \in \{0,1,-1\}^{n\times|E|}$ defined as:

$$B_{ke} = \begin{cases} 1 & \exists l, \quad \text{s.t. } e = \{k,l\} \in G; \\ -1 & \exists l, \quad \text{s.t. } e = \{l,k\} \in G; \\ 0 & \text{otherwise.} \end{cases}$$

Then we have

$$\frac{p_j\left(t\right) + p_i\left(t\right)}{2} = \left(\boldsymbol{A}^T \cdot \frac{\boldsymbol{p}\left(t\right)}{2}\right)_{e=\{i,j\}},$$

$$\frac{p_j\left(t\right) - p_i\left(t\right)}{2} = \left(\boldsymbol{B}^T \cdot \frac{\boldsymbol{p}\left(t\right)}{2}\right)_{e=\{i,j\}},$$

$$F_j\left(\boldsymbol{p}\left(t\right)\right) - F_i\left(\boldsymbol{p}\left(t\right)\right) = \left(\boldsymbol{B}^T \cdot \boldsymbol{F}\right)_{e=\{i,j\}},$$

where $\boldsymbol{F} = \boldsymbol{F}(\boldsymbol{\theta}, \boldsymbol{p}(t)) = \boldsymbol{\Phi} + \boldsymbol{W} \cdot \boldsymbol{p}(t) + \beta \log \boldsymbol{p}(t)$.

Therefore, we can write the system Equation (2) as

$$\frac{dp_i(t)}{dt} = -\left(\boldsymbol{B} \cdot \boldsymbol{E}(\boldsymbol{\theta}, \boldsymbol{p}(t))\right)_i,$$

where $\boldsymbol{E}(\boldsymbol{\theta}, \boldsymbol{p}(t)) = \left(\boldsymbol{A}^T \cdot \frac{\boldsymbol{p}(t)}{2}\right) \circ \left(\boldsymbol{B}^T \cdot \boldsymbol{F}\right) + \left(\boldsymbol{B}^T \cdot \frac{\boldsymbol{p}(t)}{2}\right) \circ \left|\boldsymbol{B}^T \cdot \boldsymbol{F}\right|$ and the operation $\circ$ is entry-wise product defined as $\boldsymbol{a} \circ \boldsymbol{b} = \left[a_1 b_1, a_2 b_2, \cdots, a_{|E|} b_{|E|}\right]^T$.

Similarly, we also write the objective function as

$$\mathcal{L}(\boldsymbol{\theta}) = \int_{t_1}^{t_f} L(\boldsymbol{\theta}, \boldsymbol{p}(t))\, dt + \sum_{l=2}^{f} \lambda_l \mathrm{KL}\left(\boldsymbol{p}(t_l) \parallel \boldsymbol{p}^l\right),$$

where $L(\boldsymbol{\theta}, \boldsymbol{p}(t)) = \frac{1}{2}\left(\boldsymbol{B}^T \cdot \boldsymbol{F}\right)^T \cdot \boldsymbol{E}(\boldsymbol{\theta}, \boldsymbol{p}(t))$.

The above minimization problem of the discrete L_2-Wasserstein distance can be further recast in the form of an optimal control problem with parameters $\boldsymbol{\theta}$ regarded as the control,

$$\inf_{\boldsymbol{\theta}} \quad \mathcal{L}(\boldsymbol{\theta}) = \int_{t_1}^{t_f} L(\boldsymbol{\theta}, \boldsymbol{p}(t))\, dt + \sum_{l=2}^{f} \lambda_l \mathrm{KL}\left(\boldsymbol{p}(t_l) \parallel \boldsymbol{p}^l\right), \tag{4}$$

subject to the constraints

$$\frac{d\boldsymbol{p}(t)}{dt} = \boldsymbol{f}(\boldsymbol{\theta}, \boldsymbol{p}(t)) = -\boldsymbol{B} \cdot \boldsymbol{E}(\boldsymbol{\theta}, \boldsymbol{p}(t)), \tag{5}$$

$$\boldsymbol{p}(t_1) = \boldsymbol{p}^1. \tag{6}$$

Here, we adopt the celebrated Pontryagin's Maximum Principle (also known as the adjoint method) [1] to solve the above optimal control problem of Equation (4). We treat the integral part and the KL divergence part on the right-hand side (R.H.S.) of Equation (4) separately, solve each of them using the adjoint method, and then combine them together through the tradeoff parameters λ_ls.

The integral part of R.H.S. of Equation (4) can be regarded as the optimal control problem with fixed terminal time but uncertain terminal state. Let $\boldsymbol{\theta}^* = \{\boldsymbol{\Phi}^*, \boldsymbol{W}^*\}$ be the optimal control and $\boldsymbol{p}^* : [t_1, t_f] \to \mathcal{P}(G)$ be the corresponding optimal state trajectory. According to Pontryagin's Maximum Principle [1], there exists an adjoint function $\boldsymbol{\mu}^* : [t_1, t_f] \to \mathbb{R}^n$ satisfying:

$$\begin{cases} \dot{\boldsymbol{\mu}} = -\boldsymbol{\mu}^T \frac{\partial \boldsymbol{f}}{\partial \boldsymbol{p}} + \frac{\partial L}{\partial \boldsymbol{p}}, \\ \boldsymbol{\mu}(t_f) = \boldsymbol{0}. \end{cases}$$

and for each fixed $t \in [t_1, t_f]$,

$$H\left(\boldsymbol{p}^*(t), \boldsymbol{\theta}^*, \boldsymbol{\mu}^*(t)\right) = \max_{\boldsymbol{\theta}} H\left(\boldsymbol{p}^*(t), \boldsymbol{\theta}, \boldsymbol{\mu}^*(t)\right). \tag{7}$$

where $H(\boldsymbol{p}(t), \boldsymbol{\theta}, \boldsymbol{\mu}(t)) = -L(\boldsymbol{\theta}, \boldsymbol{p}(t)) + \boldsymbol{\mu}^T(t) \cdot \boldsymbol{f}(\theta, \boldsymbol{p}(t))$.

From Equation (7), once $\boldsymbol{p}^*$, $\boldsymbol{u}^*$ are already known, we can get the necessary conditions that $\boldsymbol{\theta}^*$ needs to meet:

$$\frac{\partial H(\boldsymbol{p}^*(t), \boldsymbol{\theta}^*, \boldsymbol{\mu}^*(t)}{\partial \boldsymbol{\theta}} = \mathbf{0}, \quad \forall t \in [t_1, t_f]$$

Notice that in our situation, control parameter $\boldsymbol{\theta}$ is constant over the entire time period, then we can derive $\boldsymbol{\theta}^*$ by the gradient ascent method:

$$\boldsymbol{\theta_{k+1}} \leftarrow \boldsymbol{\theta_k} + \alpha \cdot \int_{t_1}^{t_f} \frac{\partial H}{\partial \boldsymbol{\theta}} (t, \boldsymbol{p}^*, \boldsymbol{\theta}^*, \boldsymbol{\mu}^*) dt.$$

In practical application, $\boldsymbol{p}^*$, $\boldsymbol{\mu}^*$ cannot be calculated in advance. However, once $\boldsymbol{\theta}$ is known, the corresponding $\boldsymbol{p}$ and $\boldsymbol{\mu}$ can be calculated. Based on their coupling relationships, we can derive $\boldsymbol{\theta}^*$ (for the integral part) by the following iterative steps:

$$\begin{aligned}
\boldsymbol{p_k}(t) &\leftarrow \boldsymbol{p}^1 + \int_{t_1}^{t} \boldsymbol{f}(\boldsymbol{\theta_k}, \boldsymbol{p_k}(\tau))\, d\tau, \\
\boldsymbol{\mu_k}(t) &\leftarrow \mathbf{0} - \int_{t}^{t_f} \left(\frac{\partial L}{\partial \boldsymbol{p}} (\boldsymbol{\theta_k}, \boldsymbol{p_k}(\tau)) - \boldsymbol{\mu}_{\boldsymbol{k}}^T(\tau) \cdot \frac{\partial \boldsymbol{f}}{\partial \boldsymbol{p}} (\boldsymbol{\theta_k}, \boldsymbol{p_k}(\tau)) \right) d\tau, \\
\boldsymbol{\theta_{k+1}} &\leftarrow \boldsymbol{\theta_k} - \alpha \int_{t_1}^{t_f} \left(\frac{\partial L}{\partial \boldsymbol{\theta}} (\boldsymbol{\theta_k}, \boldsymbol{p_k}(\tau)) - \boldsymbol{\mu_k}^T(\tau) \cdot \frac{\partial \boldsymbol{f}}{\partial \boldsymbol{\theta}} (\boldsymbol{\theta_k}, \boldsymbol{p_k}(\tau)) \right) d\tau,
\end{aligned}$$

where subscript $\boldsymbol{k}$ in bold indicates the k-th iteration, and α is the learning rate.

For the KL divergence part of R.H.S. of Equation (4), NeuralODE [2] introduced an adjoint function $\boldsymbol{a} : [t_1, t_f] \to \mathbb{R}^n$ and proved that

$$\frac{\mathrm{dKL}\left(\boldsymbol{p}^*(t_l) \parallel \boldsymbol{p}^l\right)}{\mathrm{d}\boldsymbol{\theta}} = \int_{t_{l-1}}^{t_l} \boldsymbol{a}^T(t) \cdot \frac{\partial \boldsymbol{f}}{\partial \boldsymbol{\theta}} dt, \tag{8}$$

where $\boldsymbol{a}(t) = \frac{\mathrm{dKL}\left(\boldsymbol{p}^*(t) \| \boldsymbol{p}^t\right)}{\mathrm{d}\boldsymbol{p}(t)}$, $\frac{\mathrm{d}\boldsymbol{a}(t)}{\mathrm{d}t} = -\boldsymbol{a}^T(t) \cdot \frac{\partial \boldsymbol{f}(\boldsymbol{\theta}, \boldsymbol{p}(t))}{\partial \boldsymbol{p}(t)}$, $\boldsymbol{p}^t$ and $\boldsymbol{p}(t)$ represent the real and optimal estimated cell type-based probability distribution at t, respectively.

Similarly, we can derive $\boldsymbol{\theta}^*$ (for the KL divergence part) by the following iterative steps:

$$\begin{aligned}
\boldsymbol{p_k}(t) &\leftarrow \boldsymbol{p}^1 + \int_{t_1}^{t} \boldsymbol{f}(\boldsymbol{\theta_k}, \boldsymbol{p_k}(\tau))\, d\tau, \\
\boldsymbol{a_k}(t) &\leftarrow \frac{\mathrm{dKL}\left(\boldsymbol{p_k}(t_f) \parallel \boldsymbol{p}^f\right)}{\mathrm{d}\boldsymbol{p}(t_f)} + \int_{t}^{t_f} \left(\boldsymbol{a}_{\boldsymbol{k}}^T(\tau) \cdot \frac{\partial \boldsymbol{f}}{\partial \boldsymbol{p}} (\boldsymbol{\theta_k}, \boldsymbol{p_k}(\tau)) \right) d\tau, \\
\boldsymbol{\theta_{k+1}} &\leftarrow \boldsymbol{\theta_k} - \alpha \sum_{l=2}^{l=f} \lambda_l \left(\int_{t_{l-1}}^{t_l} \boldsymbol{a_k}^T(\tau) \cdot \frac{\partial \boldsymbol{f}}{\partial \boldsymbol{\theta}} (\boldsymbol{\theta_k}, \boldsymbol{p_k}(\tau)) d\tau \right),
\end{aligned}$$

where subscript $\boldsymbol{k}$ in bold indicates the k-th iteration, and α is the learning rate. We follow NeuralODE [2] and approximate $\{\boldsymbol{a_k}(t)\}_{t \in [t_1, t_f]}$ as follows:

$$\boldsymbol{a_k}(t) \approx \boldsymbol{a_k}(t_{l+1}) = \frac{d\mathrm{KL}\left(\boldsymbol{p_k}(t_{l+1}) | \boldsymbol{p}^{l+1}\right)}{d\boldsymbol{p_k}(t_{l+1})} \quad \forall t \in [t_l, t_{l+1}].$$

We also approximate $\{\boldsymbol{\mu}(t)\}_{t\in[t_l,t_{l+1}]}$ as $\boldsymbol{\mu}(t_l)$ in the same approach.

Putting these together, we estimate $\boldsymbol{\theta}^*$ by combining the two optimization parts in the following iterative steps:

$$
\begin{aligned}
&\boldsymbol{p_k}(t) \leftarrow \boldsymbol{p}^1 + \int_{t_1}^{t} \boldsymbol{f}\left(\boldsymbol{\theta_k}, \boldsymbol{p_k}(\tau)\right) d\tau,\\
&\boldsymbol{\mu_k}(t) \leftarrow \text{ODESolve}\left(\mathbf{0}, \frac{\partial L}{\partial \boldsymbol{p}}\left(\boldsymbol{\theta_k}, \boldsymbol{p_k}\right) - \boldsymbol{\mu}_{\boldsymbol{k}}^T \cdot \frac{\partial \boldsymbol{f}}{\partial \boldsymbol{p}}\left(\boldsymbol{\theta_k}, \boldsymbol{p_k}\right), t_f, t_l\right), \quad \forall t \in [t_l, t_{l+1}],\\
&\boldsymbol{a_k}(t) \leftarrow \frac{\text{dKL}\left(\boldsymbol{p_k}\left(t_{l+1}\right) \parallel \boldsymbol{p}^{l+1}\right)}{\text{d}\boldsymbol{p}\left(t_{l+1}\right)}, \quad \forall t \in [t_l, t_{l+1}],\\
&\boldsymbol{\theta_{k+1}} \leftarrow \boldsymbol{\theta_k} - \alpha \sum_{l=2}^{l=f} \int_{t_{l-1}}^{t_l} \Big[\left(\lambda_l \boldsymbol{a}_{\boldsymbol{k}}^T(\tau) - \boldsymbol{\mu}_{\boldsymbol{k}}^T(\tau)\right) \cdot \frac{\partial \boldsymbol{f}}{\partial \boldsymbol{\theta}}\left(\boldsymbol{\theta_k}, \boldsymbol{p_k}(\tau)\right) + \frac{\partial L}{\partial \boldsymbol{\theta}}\left(\boldsymbol{\theta_k}, \boldsymbol{p_k}(\tau)\right) \Big] d\tau,
\end{aligned}
$$

where subscript $\boldsymbol{k}$ in bold indicates the k-th iteration, and α is the learning rate. Here, the *ODESolve* function is implemented by the *deSolve* package of R [3], a solver for initial value problems of differential equations.

II: Derive forms for the gradients in the iteration process of GraphFP

$$
\begin{aligned}
\frac{\partial \boldsymbol{f}}{\partial \boldsymbol{\Phi}} &= -\boldsymbol{B}\left[\boldsymbol{q} \circ \boldsymbol{B}^T\right]\\
\frac{\partial f_i}{\partial \boldsymbol{W}} &= \left[\boldsymbol{p}\frac{\partial f_i}{\partial \boldsymbol{\Phi}}\right]^T\\
\frac{\partial \boldsymbol{f}}{\partial \boldsymbol{p}} &= -\boldsymbol{B}\left[\left(\boldsymbol{q} \circ \boldsymbol{B}^T\right)\frac{\partial \boldsymbol{F}}{\partial \boldsymbol{p}} + \left(\boldsymbol{B}^T\boldsymbol{F}\right) \circ \left(\frac{\boldsymbol{A}^T + \boldsymbol{B}^T}{2}\right)\right]\\
\frac{\partial L}{\partial \boldsymbol{\Phi}} &= -\left[\frac{\partial \boldsymbol{f}}{\partial \boldsymbol{\Phi}}\boldsymbol{F}\right]^T\\
\frac{\partial L}{\partial \boldsymbol{W}} &= \left[\boldsymbol{p}\frac{\partial L}{\partial \boldsymbol{\Phi}}\right]^T\\
\frac{\partial L}{\partial \boldsymbol{p}} &= -\frac{1}{2}\boldsymbol{F}^T\frac{\partial \boldsymbol{f}}{\partial \boldsymbol{p}} + \frac{1}{2}\left[\left(\boldsymbol{q} \circ \boldsymbol{B}^T\right)\boldsymbol{F}\right]^T \circ \left(\boldsymbol{B}^T\frac{\partial \boldsymbol{F}}{\partial \boldsymbol{p}}\right)
\end{aligned}
$$

where

$$
\begin{aligned}
&\boldsymbol{a} \circ \boldsymbol{b} \equiv \{a_1b_1, a_2b_2, \cdots, a_{|E|}b_{|E|}\}^T, \quad \forall \boldsymbol{a}, \boldsymbol{b} \in \mathbb{R}^{|E|\times 1}\\
&\boldsymbol{a} \circ \boldsymbol{M} \equiv \begin{pmatrix} a_1m_{11} & a_1m_{12} & \cdots & a_1m_{1n}\\ a_2m_{21} & a_2m_{22} & \cdots & a_2m_{2n}\\ \vdots & \vdots & \vdots & \vdots\\ a_{|E|}m_{|E|1} & a_{|E|}m_{|E|2} & \cdots & a_{|E|}m_{|E|n} \end{pmatrix}, \quad \forall \boldsymbol{a} \in \mathbb{R}^{|E|\times 1}, \boldsymbol{M} \in \mathbb{R}^{|E|\times n},
\end{aligned}
$$

$$\boldsymbol{q} = \boldsymbol{A}^T \frac{\boldsymbol{p}}{2} + \boldsymbol{B}^T \frac{\boldsymbol{p}}{2} \circ \operatorname{sign}\left(\boldsymbol{B}^T \boldsymbol{F}\right) \in \mathbb{R}^{|E| \times 1}$$

$$\frac{\partial \boldsymbol{F}}{\partial \boldsymbol{p}} = \boldsymbol{W} + \beta \begin{pmatrix} \frac{1}{p_1} & 0 & \cdots & 0 \\ 0 & \frac{1}{p_2} & \cdots & 0 \\ \vdots & \vdots & \vdots & \vdots \\ 0 & 0 & \cdots & \frac{1}{p_n} \end{pmatrix}$$

III: Pseudocode for the GraphFP Algorithm

Algorithm A The Algorithm of GraphFP

1: **Input:** Time series data with cell type annotation and time annotation; The total number of cell types n; Learning rate α; Regularization parameters $\{\lambda_l\}_{l=2}^{f}$ and β; Integral Step Δt.
2: // 5-15 for solving GraphFP problem
3: Represent system state at t_l as proportions of cell types according to cell type annotation and time annotation, $\{\boldsymbol{p^1}, \boldsymbol{p^2}, \cdots, \boldsymbol{p^f}\}$.
4: Construct cell state-transition graph G (a complete graph of n vertices for default).
5: Initialize $\boldsymbol{\theta_0} = \{\boldsymbol{\Phi_0}, \boldsymbol{W_0}\} \leftarrow \{\boldsymbol{0}, \boldsymbol{0}\}; \boldsymbol{k} \leftarrow 0$
6: **while** not converged **do**
7:     // Calculate the corresponding state process $\boldsymbol{p_k}$ of $\boldsymbol{\theta_k}$
8:     $\boldsymbol{p_k}(t) \leftarrow \boldsymbol{p}^1 + \int_{t_1}^{t} \boldsymbol{f}\left(\boldsymbol{\theta_k}, \boldsymbol{p_k}(\tau)\right) d\tau$
9:     // Calculate adjoint variables $\boldsymbol{u_k}, \boldsymbol{a_k}$ based on $\boldsymbol{\theta_k}$
10:     $\boldsymbol{\mu_k}(t) \leftarrow \text{ODESolve}\left(\boldsymbol{0}, \frac{\partial L}{\partial \boldsymbol{p}}\left(\boldsymbol{\theta_k}, \boldsymbol{p_k}\right) - \boldsymbol{\mu_k}^T \cdot \frac{\partial \boldsymbol{f}}{\partial \boldsymbol{p}}\left(\boldsymbol{\theta_k}, \boldsymbol{p_k}\right), t_f, t_l\right), \quad \forall t \in [t_l, t_{l+1}],$
11:     $\boldsymbol{a_k}(t) \leftarrow \frac{\mathrm{dKL}\left(\boldsymbol{p_k}(t_{l+1}) \| \boldsymbol{p}^{l+1}\right)}{\mathrm{d}\boldsymbol{p}(t_{l+1})}, \quad \forall t \in [t_l, t_{l+1}],$
12:     // Update $\boldsymbol{\theta_k}$ to $\boldsymbol{\theta_{k+1}}$
13:     $\boldsymbol{\theta_{k+1}} \leftarrow \boldsymbol{\theta_k} - \alpha \sum_{l=2}^{l=f} \int_{t_{l-1}}^{t_l} \left[\left(\lambda_l \boldsymbol{a_k}^T(\tau) - \boldsymbol{\mu_k}^T(\tau)\right) \cdot \frac{\partial \boldsymbol{f}}{\partial \boldsymbol{\theta}}\left(\boldsymbol{\theta_k}, \boldsymbol{p_k}(\tau)\right) + \frac{\partial L}{\partial \boldsymbol{\theta}}\left(\boldsymbol{\theta_k}, \boldsymbol{p_k}(\tau)\right) \right] d\tau$
14:     $\boldsymbol{k} \leftarrow \boldsymbol{k} + \boldsymbol{1}$
15: **return** $\boldsymbol{\theta_k} = \{\boldsymbol{\Phi_k}, \boldsymbol{W_k})$

References

[1] Arthur E Bryson and YuChi Ho. *Applied Optimal Control: Optimization, Estimation and Control*. CRC Press, 1975.

[2] Ricky T. Q. Chen, Yulia Rubanova, Jesse Bettencourt, and David K Duvenaud. Neural ordinary differential equations. In S. Bengio, H. Wallach, H. Larochelle, K. Grauman, N. Cesa-Bianchi, and R. Garnett, editors, *Advances in Neural Information Processing Systems*, volume 31, pages 6571–6583. Curran Associates, Inc., 2018.

[3] Karline Soetaert, Thomas Petzoldt, and R. Woodrow Setzer. Solving differential equations in R: Package deSolve. *Journal of Statistical Software*, 33(9):1–25, 2010.
