# Supplementary material for: Dynamic inference of cell developmental complex energy landscape from time series single-cell transcriptomic data
Source: PLoS Comput Biol. 2022 Jan 24;18(1):e1009821. doi: 10.1371/journal.pcbi.1009821 (PMC8812873; doi:10.1371/journal.pcbi.1009821)
Supplement: S2 Text — Fig A. GraphFP reconstructs the cell state-transition energy landscape on the mouse spinal cord injury scRNA-seq dataset. Fig B. The linear potential energy Φ quantifies cell differentiation potency. Table A. Evaluation of GraphFP’s performance on quantifying the stochastic dynamics of cell type frequencies with cell-cell interaction term (W ≠ 0) and without cell-cell interaction term (W = 0) on the mouse spinal cord injury dataset. (PDF) [file pcbi.1009821.s002.pdf]

Dynamic inference of cell developmental complex energy landscape from time series single-cell transcriptomic data

Qi Jiang, Shuo Zhang, Lin Wan

Supporting Information
S2 Text. Application of GraphFP to the mouse spinal cord injury dataset

We further analyzed a time series scRNA-seq dataset of the mouse spinal cord injury healing process provided by [1]. The time series transcriptomic profile contains 66,176 cells collected after spinal cord injury over four time points, including the uninjured and injured spinal cord at 1, 3, and 7 day post-injury (dpi). In our implementation of GraphFP, we set the uninjured samples as time point 0 dpi. The cells of the mouse spinal cord injury dataset were clustered into 15 cell types in [1]. These 15 clusters represent all major cell types that are known to comprise the spinal cord injury site including "1-Astrocyte", "2-Dendritic", "3-Div-Myeloid", "4-Endothelial", "5-Ependymal", "6-Fibroblast", "7-Macrophage", "8-Microglia", "9-Monocyte", "10-Neutrophil", "11-Oligodendrocyte", "12-OPC", "13-Pericyte", "14-Neurons" and "15-Lymphocytes". In our implementation, we followed [1] to remove "14-Neurons" and "15-Lymphocytes", resulting in 13 cell types remained for our further analysis.

Using the same hyper-parameter settings as those for the murine cerebral cortex dataset, GraphFP accurately reconstructed the cell state-transition energy landscape of the mouse spinal cord injury dataset (Fig A).

GraphFP estimated parameters $\boldsymbol{\theta} = \{\boldsymbol{\Phi}, \boldsymbol{W}\}$ of the free energy (Fig A(a) and A(b)). In general, the static landscape of the estimated linear potential energy $\boldsymbol{\Phi}$ shows a strong correlation with the trend of probability curves over time (Fig A(a) and A(e)). Specifically, cell types whose probability decreases over time have positive Φs, such as "4-Endothelial" ($\Phi_4 = 0.039$), "5-Ependymal" ($\Phi_5 = 0.037$), "11-Oligodendrocyte" ($\Phi_{11} = 0.012$), "12-OPC" ($\Phi_{12} = 0.016$) and "13-Pericyte" ($\Phi_{13} = 0.023$). Cell types whose probability increases over time have negative Φs (Fig A(a) and A(e)), such as "8-Microglia" ($\Phi_8 = -0.015$).

We further demonstrated that the linear potential $\boldsymbol{\Phi}$ estimated by GraphFP can be used to quantify the cell differentiation potency. We assigned each cell with the same linear potential value as that of its corresponding cell type/state. Firstly, we tested whether our linear potential energies for pluripotent stem cells (at early time point) are higher than those for differentiated cells (at latter time point). It is clearly shown that, cells from samples collected at earlier time stages tend to have higher potential Φ and vice versa (Fig B). When using the one-sided Wilcoxon ranksum statistic as applied by Shi *et al.* [2], we confirmed with highly statistically significant results that the linear potential values of cells sampled at the earliest time stage 0 dpi are higher than those cells sampled at the subsequent time stages 1 dpi ($p < 2.2e-16$), 3 dpi ($p < 2.2e-16$), and 7 dpi ($p < 2.2e-16$), respectively.

The estimated $\boldsymbol{W}$ is a sparse matrix with most elements having values close to zero (Fig A(b)). The values of the rows corresponding to "1-Astrocyte" and "13-Pericyte" are zero or close to zero with $|w_{ij}| \leq 0.03$, suggesting that no other cell types have significant influence on them. As such, their potential energies $\Psi_1(t)$ and $\Psi_{13}(t)$ are dominated by their linear potential energies (Φ_1 and Φ_{13}) with resultant flattening potential energy curves (Fig A(c)).

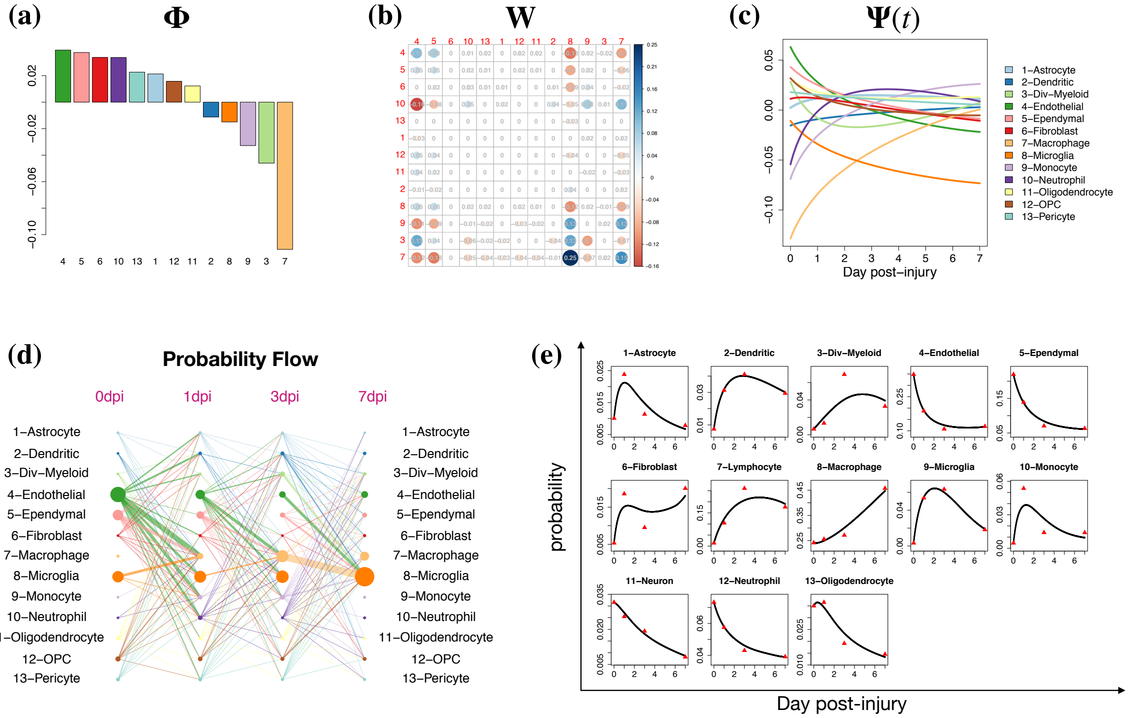

Fig A. **GraphFP reconstructs the cell state-transition energy landscape on the mouse spinal cord injury scRNA-seq dataset.** **(a)** The estimated linear potential energy $\mathbf{\Phi}$. **(b)** The estimated cell-cell interaction matrix $\boldsymbol{W}$. **(c)** The reconstructed potential energy landscape $\mathbf{\Psi}(t)$ of cell types (colored curves) over time. **(d)** GraphFP charted the probability flow of cell state-transitions: circle point represents cell type (point size is proportional to the cell type frequency at each time point); the line between cell types represents probability flow from source cell type to target cell type (line width is proportional to the value of probability flow). **(e)** GraphFP quantified the stochastic dynamics of cell type frequencies $\boldsymbol{p}(t)$ on probability simplex in continuous time.

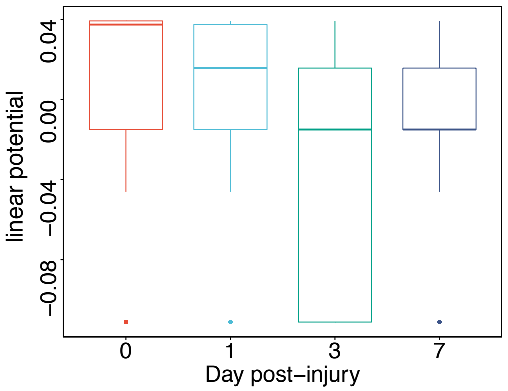

Fig B. **The linear potential energy $\mathbf{\Phi}$ quantifies cell differentiation potency.** Boxplot of the linear potential energies of cells sampled different time stages of the mouse spinal cord injury healing process.

We also observed a number of strong cell-cell interactions with large w_{ij}s deviating from zero. The values of the rows corresponding to "7-macrophage" and "8-Microglia" are almost deviating from zero, resulting a sharpened potential energy curve (Fig A(c)). Furthermore, the potential energy curve of "8-Microglia" shows a decreasing trend over time, indicating that "8-Microglia" becomes more stable and more attractive to other cell types to transit to "8-Microglia".

In addition, GraphFP accurately quantified the stochastic dynamics of the cell type frequencies (Fig A(e)), especially for the complex trend of "6-Fibroblast". To show the power of the cell-cell interaction term for quantifying the stochastic dynamics, we evaluated GraphFP on its ability to fit the experimental data and recover held-out time points with cell-cell interaction term and without cell-cell interaction term. To evaluate the performance on estimation accuracy, we applied Kullback-Leibler divergence (KLD) to measure the difference between the estimated probability distribution by GraphFP with/without interaction term and true probability distribution at each time points (Table A). A lower KLD value is indicative of better performance. From Table A, GraphFP with cell-cell interaction term always achieved better estimations with higher accuacy for observed time points (1dpi, 3dpi, 7dpi) over GraphFP without cell-cell interaction term, whether taking all time points into training or holding out the intermediate time points. Overall, GraphFP with cell-cell interaction term outperforms GraphFP without cell-cell interaction term.

Table A. **Evaluation of GraphFP's performance on quantifying the stochastic dynamics of cell-type frequencies with cell-cell interaction term ($\boldsymbol{W} \neq 0$) and without cell-cell interaction term ($\boldsymbol{W} = 0$) on the mouse spinal cord injury dataset.**

**KLD**	**Using all time points**		**Held out 1dpi**		**Held out 3dpi**		**Held out 1dpi and 3dpi**	
	**with**	**without**	**with**	**without**	**with**	**without**	**with**	**without**
1dpi	**0.0060**	0.1285	**0.0535**	0.1312	**0.0002**	0.1486	**0.0930**	0.1606
3dpi	**0.0215**	0.0934	**0.0137**	0.07934	**0.0957**	0.1689	**0.1513**	0.2021
7dpi	**0.0033**	0.2472	**0.0133**	0.2654	**2.0969e-05**	0.1942	**6.1153e-11**	0.1883

The Kullback-Leibler divergence (KLD) distance was used to measure the difference between the estimated probability distribution by GraphFP and true probability distribution at each time points (1dpi, 3dpi, 7dpi).

References

[1] Lindsay M. Milich, James S. Choi, Christine Ryan, Susana R. Cerqueira, Sofia Benavides, Stephanie L. Yahn, Pantelis Tsoulfas, and Jae K. Lee. Single-cell analysis of the cellular heterogeneity and interactions in the injured mouse spinal cord. *Journal of Experimental Medicine*, 218(8), 06 2021.

[2] Jifan Shi, Tiejun Li, Luonan Chen, and Kazuyuki Aihara. Quantifying pluripotency landscape of cell differentiation from scRNA-seq data by continuous birth-death process. *PLOS Computational Biology*, 15(11):1–17, 11 2019.
