# Supplementary material for: Dynamic inference of cell developmental complex energy landscape from time series single-cell transcriptomic data
Source: PLoS Comput Biol. 2022 Jan 24;18(1):e1009821. doi: 10.1371/journal.pcbi.1009821 (PMC8812873; doi:10.1371/journal.pcbi.1009821)
Supplement: S1 Table — (DOCX) [file pcbi.1009821.s003.docx]

**S1 Table. Runtimes of GraphFP with cell-cell interaction term and without cell-cell interaction term on the murine cerebral cortex dataset.**

| **Runtime** | **Using all time points** | | **Held out E13.5** | | **Held out E15.5** | | **Held out E13.5 and E15.5** | |
| --- | --- | --- | --- | --- | --- | --- | --- | --- |
|  | **with** | **without** | **with** | **without** | **with** | **without** | **with** | **without** |
|  | 2.7 mins | 8.7 secs | 3.1 mins | 3.3 secs | 3.1 mins | 9.1 secs | 11.3 secs | 10.0 secs |

The computation is performed on a MacBook Pro laptop with CUP 2.4 GHz Intel Core i5 and Memory 8 GB 2133 MHz LPDDR3.
