# Supplementary material for: Dynamic inference of cell developmental complex energy landscape from time series single-cell transcriptomic data
Source: PLoS Comput Biol. 2022 Jan 24;18(1):e1009821. doi: 10.1371/journal.pcbi.1009821 (PMC8812873; doi:10.1371/journal.pcbi.1009821)
Supplement: S2 Table — (DOCX) [file pcbi.1009821.s004.docx]

**S2 Table. Runtimes of GraphFP with cell-cell interaction term and without cell-cell interaction term on the mouse spinal cord injury dataset.**

| **Runtime** | **Using all time points** | | **Held out 1dpi** | | **Held out 3dpi** | | **Held out 1dpi and 3dpi** | |
| --- | --- | --- | --- | --- | --- | --- | --- | --- |
|  | **with** | **without** | **with** | **without** | **with** | **without** | **with** | **without** |
|  | 9.1 mins | 4.8 secs | 2.0 mins | 4.6 secs | 10.4 mins | 4.9 secs | 1.3 mins | 16.2 secs |

The computation is performed on a MacBook Pro laptop with CUP 2.4 GHz Intel Core i5 and Memory 8 GB 2133 MHz LPDDR3.
