# Supplementary material for: Dynamic inference of cell developmental complex energy landscape from time series single-cell transcriptomic data
Source: PLoS Comput Biol. 2022 Jan 24;18(1):e1009821. doi: 10.1371/journal.pcbi.1009821 (PMC8812873; doi:10.1371/journal.pcbi.1009821)
Supplement: Attachment — Submitted filename: ResponseLetter-GraphFP.pdf [file pcbi.1009821.s005.pdf]

Responses to Reviewers:

We would like to thank the reviewers for thoughtful comments and valuable suggestions on our manuscript. The manuscript has been fully revised by following the reviewers' comments and suggestions, making our results more solid and convincing.

The point-by-point responses to the reviewers' comments and suggestions are as follows and our responses are in red color.

---

Reviewer #1: In this work, the authors propose an approach to infer cell state transition dynamics and uncover cell-cell interactions from time series single-cell RNA-seq data, by constructing the energy landscape of the system. Based on a nonlinear Fokker-Planck equation on Graph based model, they solve the model inference problem in a dynamic optimal transport framework. They further illustrate the validity of their approach by applying it to a time series scRNA-seq data set of embryonic murine cerebral cortex development.

Overall, this is an interesting work, aiming to address a critical problem in dynamic model inference and data-driven approach in biology. However, I have some concerns that need to be addressed.

1, The authors assume that the cell-cell interaction matrix W is symmetric, what does this assumption mean in biology? And why is this a reasonable assumption? For example, should cells with different differentiation potency have symmetric interactions?

Reply: Thank you for the insightful comments. We agree with you that the cell-cell interaction strengths between two cell types do not need to be symmetric. In the revised manuscript, we have removed the symmetry constraint on W, and provided the biological interpretation on W, as follows:

1) We removed the symmetry constraint on W:
"In general, the interaction matrix W is asymmetry, with its element w_ij representing the interaction strength from cell state j (as a signalling sender) to cell state i (as a signalling receiver) (see Eq (9) and section "Reconstruction of cell developmental energy landscape and modelling of cell-cell interactions" for further discussion)." (Lines 132-136 on page 5 of the main text).

2) We interpreted the biological meaning of the cell-cell interaction matrix W in the new section "Reconstruction of cell developmental energy landscape and modelling of cell-cell interactions" as follows:
"Cell development relies on the coordination of cellular activities based on temporal and local cell-cell communication through molecular signalling events [22]. As a key component, the interaction potential energy $\sum_{k=1}^{n} w_{ik} p_k(t)$ in Eq (8) models and quantifies cell-cell interactions that drive cell development, where w_ik is the interaction strength from cell type k (as a signalling sender) to cell type i (as a signalling receiver): when w_ik > 0, cell type k will send signals to upgrade the potential energy of cell type i to a higher level, thus inhibiting the decrease of potential energy of cell type i; when w_ik < 0, cell type k will send signals to downgrade the potential energy of cell type i to a lower level, thus stimulating the decrease of potential energy of cell type i; when w_ik = 0 or w_ik ≈ 0, cell type k will send no or weak signals to cell type i, thus unable alter the potential energy level of cell type i. We say

that cell types i and k have no mutual interactions when both w_ik and w_ki are zero or close to zero." (Lines 178-190 on page 7 of the main text).

3) We provided the literatures to support our assumptions on the new W as follows: "Such modelling of the interaction matrix W is inspired and evidenced by our increasing understanding that both positive and negative feedback circuits composed of both stimulatory and inhibitory factors are involved in the regulation of precise coordination of cell fate decisions through intercellular communication [22,30]." (Lines 190-194 on pages 7-8 of the main text).

4) We recalculated all the results on the new W, and updated these results in the revised manuscript. We achieved consistent results as the previous version, except for part on estimated new W.

2, Fig.2 (a)(b)(c), the use of color for different cell types is very confusing. Please try to make them consistent.

Reply: Thank you for pointing out this confusion. In the revised manuscript, we have modified the colors in Fig 2(a) to make it be consistent with those shown in Fig 2(b, c).

3, It's interesting to get cell-cell interaction matrix W as in Fig.2. However, the biological indication needs to be further discussed. For example, from Fig. 2 (g and h) it is found that cell type 3 (Aps/RPs) has positive interaction with cell type 6 (Young Neurons), and negative interaction with cell type 4 (IPs) or 2 (Young Neurons). What could be the biological meaning here based on the linage commitment route in Fig. 2(a)? Why do the two Young Neurons populations (cell type 6 and 2) have opposite types of interactions with cell type 3 (Aps/RPs)?

Reply: Thank you for your valuable suggestion. In the revised manuscript, in response to the Comment 1 above, we removed the symmetric constraint on the cell-cell interaction matrix W, provided the biological meaning of W, and recalculated all results (See our response to Comment 1 above). Under the new W defined, the estimated W is biological interpretable, and the results are consistent with the linage commitment route in Fig 2(a).

In the revised manuscript, we summarized the major results on W in a newly added section "GraphFP delineates cell-cell interactions" (on pages 11, 13 of the main text) and also demonstrated that dynamic potential energy Ψ(t) with an interaction energy part provides a global and holistic view of cell development process (see the example of "2-Young Neurons" and "1-Neurons" at Lines 240-258 on page 10 of the main text).

To be specific, we explored the biological indications of W as follows:

1) "The estimated W is a sparse matrix with most elements having values equal or close to zero (Fig 2(d)), indicating a majority of the pairs having no or weak interactions. For example, the cell types "5-APs/RPs" and "7-IPs" have no mutual interactions with w_57 = 0 and w_75 = −0.02 (Fig 2(d)). Furthermore, the estimated cell-cell interaction strengths in the first row of W that corresponds to "5-APs/RPs" are zero or close to zero with |w_ij| ≤ 0.04, making the contributions from its interaction term in Eq (8) negligible. As such, the dynamic potential energy Ψ5(t) is dominated by its linear potential energy (Φ5) with a resultant flattening potential energy curve (Fig 2(g)). Cell states other than "5-APs/RPs" and "7-IPs" have at least one w_ij with strong interaction strength (e.g., |w_ij| > 0.1), resulting

in the deviation of their potential energies Ψ(t)s from their linear potential energies Φs largely driven by their interaction energies with sharpened potential energy curves (Fig 2(g)).” (Lines 307-319 on page 11 of the main text).

2) “The pairs of “2-Young Neurons ← 1-Neurons” ($w\_21 = 0.26$), “6-Young Neurons ← 3-APs/RPs” ($w\_63 = 0.14$) and “4-IPs ← 1-Neurons” ($w\_41 = 0.12$) have the top 3 highest positive values of w_ijs (Fig 2(d)), indicating that the sender cell types (“1-Neurons”, “3-APs/RPs” and “1-Neurons”) pass strong inhibitory signalling to their corresponding receiver cell types (“2-Young Neurons”, “6-Young Neurons” and “4-IPs”, respectively). Their potential energy differences (|Δ_ij|s) diverge with enlarging gaps as time evolves (Fig 2(h)), resulting in that cells tend to transit in one direction from the cell state with higher potential energy to the cell state with lower potential energy, only rarely transiting in the reverse direction. In particular, “2-Young Neurons” tends to transit to “1-Neurons”, “3-APs/RPs” tends to transit to “6-Young Neurons”, “4-IPs” tends to transit to “1-Neurons”. These results are consistent with our understanding of the cell development process depicted in Fig 2(a).” (Lines 320-322 on page 11 of the main text; Lines 323-332 on page 13 of the main text).

3) “On the other hand, the pairs of “6-Young Neurons ← 1-Neurons” ($w\_61 = -0.21$), “4-IPs ← 3-APs/RPs” ($w\_43 = -0.15$) and “2-Young Neurons ← 4-IPs” ($w\_24 = -0.14$) have the top 3 lowest negative values of w_ijs, indicating that the sender cell types (“1-Neurons”, “3-APs/RPs” and “4-IPs”) pass strong stimulatory signalling to their receiver cell types (“6-Young Neurons”, “4-IPs” and “2-Young Neurons”, respectively). In particular, the potential energy differences (|Δ_ij|s) of the pair “6-Young Neurons : 1-Neurons” and the pair “3-APs/RPs : 4-IPs” converge with shrinking gaps as time evolves (Fig 2(i)), making the transitions between the paired cell states approaching to equilibrium in both directions. This result is consistent with our probability flow (Fig 4), where the net probability flows from “6-Young Neurons” to “1-Neurons” as well as from “3-APs/RPs” to “4-IPs” gradually decrease over time. The potential energy difference between “4-IPs” and “2-Young Neurons” starts from a small value close to zero at time point E11.5, then gradually increases to its largest gap at E14, and then gradually declines to zero again at E17.5. This result indicates that the transition from “4-IPs” to “2-Young Neurons” mainly occurs at the intermediate time region from E13.5 to E15.5, which is consistent with our understanding that the “4-IPs” cells are the intermediate progenitors of cell development (Fig 2(a)).” (Lines 333-349 on page 13 of the main text).

4) “when only looking at the static landscape of Φ, we found that the linear potential energy of “1-Neurons” ($\Phi 1 = -0.073$) is slightly higher than that of “2-Young Neurons” ($\Phi 2 = -0.075$). This is in conflict with our understanding that “1-Neurons” should have the lowest potential since it is located at the terminal node of the cell lineage (Fig 2(a)). However, when looking at the dynamic potential energy Ψ(t), we found that “1-Neurons” has a strong inhibitory interaction over “2-Young Neurons” ($w\_21 = 0.26$ versus $w\_12 = 0.04$) which can upgrade the potential energy of “2-Young Neurons” during the process. The resultant dynamic potential energy of “2-Young Neurons” ($\Psi 2(t)$) surpasses that of “1-Neurons” ($\Psi 1(t)$) with higher value after time point E13 (Fig 2(g) and Left Panel of Fig 2(h)). The potential energy difference ($\Delta 21$) between “2-Young Neurons” and “1-Neurons” diverges with enlarging gaps as time evolves, especially in the latter time stages after time point E15.5 (Left Panel of Fig 2(h)). This is well consistent with our understanding that: (1) “2-Young Neurons” tends to transit to “1-Neurons” during cell development (Fig 2(a)), and (2) the transition from “2-Young Neurons” to “1-Neurons” mainly occurs at the late neurogenesis between E15.5 and E17.5 [26].” (Lines 243-258 on page 10 of the main text).

4, The energy landscape here is defined for each cell type, could it be expanded to single cell? i.e., each cell has a potential energy?

Reply: Thank you for the constructive suggestion. We agree with you that expanding the framework to single cell level is promising. However, this task is still challenging as the single cell gene expression profiles are of high error rates and high dropout rates. The pioneer work by Shi et al. [5] in this field also worked at cell cluster level to in order to achieve more accurate and robust results.

Therefore, in practice, we assigned each single cell with the same linear potential value as that of its corresponding cell type/state in the revised manuscript, and showed that reliable results were achieved based on this approach (section "The linear potential energy Φ by GraphFP quantifies cell differentiation potency" on pages 10-11 of the main text and Fig 3 on page 12 of the main text).

In the revised manuscript, we addressed this issue as follows:

"In this study, each cell will be assigned the same linear potential value as that of its corresponding cell type/state. Shi et al. [5] also quantified the cell differentiation potency at the cluster level (e.g., cell state and cell type), which makes the results more accurate and robust. Quantifying the cell differentiation potency at single-cell level is still difficult, as the single-cell gene expression profiles are known to be error-prone due to various technique issues [31]." (Lines 268-273 on pages 10-11 of the main text).

5, Current work focus on the cell-cell interactions, and molecular expression data is used only for estimating probability of cell types at different time point. As we know, the molecular interactions are important to cell fate transition determinations, and corresponding landscape construction approaches have been proposed (Kang, Advanced Science, 8, 2003133 (2021)). The authors may want to discuss the relationship between the two types of approaches.

Reply: Thank you for the insightful comment. In the revised manuscript, we discussed the two types of approaches in the Discussion part as follows:

"Modelling of cell development has long been a key goal of systems biology. The Waddington landscape is a classic metaphor for describing cell development. Mathematical framework of cell developmental energy landscape has been developed to study the dynamics of cell state-transitions from gene regulatory network (GRN) based perspective (e.g., [33–36]) and state manifold based perspective (e.g., [5,37,38]) (see [18] for a recent review of the two approaches). Traditional GRN-based landscape can be hindered by the computational issue raised by high-dimensional GRNs. Recently, a model-based dimension reduction approach of the landscape (DRL) was proposed to construct a low-dimensional energy landscape of high-dimensional GRNs [36], which overcomes the limitations of traditional methods. Although great success has been achieved, the GRN-based landscape depends on prior biological knowledge of the underlying GRN. When the information of GRNs is unavailable or not complete, the state manifold based landscape will be constructed, especially for scRNA-seq data analysis. The state manifold based methods model the cell development with stochastic Markov process and/or drift-diffusion PDE, where cell states (e.g., cell types and cell clusters) represent the local attractors of the underlying dynamic systems [37].

In this study, we propose GraphFP, a state manifold based computational framework, to reconstruct the complex potential energy landscape and infer the stochastic dynamics of cell state-transition during cell development…." (See Lines 468-497 on page 18 of the main text).

Reviewer #2: The authors consider the dynamic inference of an evolving cell population from the time series scRNA-seq data. The main idea of their approach is to apply the optimal transport theory on graph with a free energy consists of the potential, mean field interaction, and the entropy terms. The parameters describing the potential and interaction terms are estimated by fitting the model to the available data. The computation applied to embryonic murine cerebral cortex development gives reasonable results. The literature review is also fair. Overall, I think the paper is interesting and present simple yet useful tool to analyze the time series scRNA-seq data.

Some major and minor points are as below.

Major:

1. The computational cost and robustness. The authors state that the method will be applied to the cell states/types in a complete graph. If it is only for cell types, which have a small number, the DOF is small and the computation cost should be light. If it is for the whole cell states, the DOF might be huge and the computation is difficult to be done. BTW, in different time points, how will the authors choose the common cell states if they do not do grouping at first? How will the different grouping affect the result?

Reply: Thanks for your insightful comments.

(1) In the revised manuscript, we addressed the problem of the computational cost and robustness by conducting further experiments on a new dataset with increased number of cell types, and demonstrated that GraphFP achieved reasonable runtime performance on moderate number of cell types. We also discussed the possible solutions of the computation for large number of cell types. We summarized these results in the newly added section "The computational cost of GraphFP" on pages 16,18 of the main text as follows:

"We examined the impact of the number of cell types (n) on the computational cost of GraphFP. When working on the murine cerebral cortex dataset with 7 cell types (n=7) and 4 time stages, the runtime of GraphFP was around 3 minutes for each task on a personal laptop (MacBook Pro with CUP 2.4 GHz Intel Core i5 and Memory 8 GB 2133 MHz LPDDR3) (Table S2). In our implementation, we set λ_l = 1000(l ∈ 2, 3, 4), β = 0.001, the learning rate as α = 0.01/λ_l and Integral step as 0.1.

We next examined GraphFP on another time series scRNA-seq dataset of the mouse spinal cord injury healing process provided by [32] (see the detailed results in Supplementary Note S3). The new dataset contains 13 clusters (cell types) and 4 time points. We applied GraphFP to this dataset on the same computer with the same hyper-parameter settings as those for the murine cerebral cortex dataset. GraphFP still achieved accurate and robust reconstruction of cell state-transition energy landscape on this dataset (see Supplementary Note S3), and also achieved a reasonable performance on computational speed: the runtime for each task was around 9 minutes (Table S3).

Based on the above experiments, we found that the computational speed of GraphFP is sustainable for tasks with moderate number of cell types. Meanwhile, the computation of GraphFP might be problematic for large n (e.g., n > 100) at current settings. As we applied the complete cell state-transition graph, the degree of freedom (e.g., the parameters of the cell-cell interaction matrix W) will grow in the order of O(n^2), making the computation difficult. However, this problem is solvable. One way to solve this problem is to take the advantage of the sparse structure of the cell-cell interaction matrix W. As we have already noted that, the estimated Ws of both the murine cerebral cortex dataset (Fig 2(d)) and the mouse spinal cord injury dataset (Fig S1(b)) are sparse. Therefore, we can solve this problem by adding

a L1 regularization term of the matrix W to the loss function to enforce W to be sparse. We plan to pursue this topic in our future work.

On the other hand, in practice, for large number of cell types, we can trade off the estimation accuracy and the information of cell-cell interactions for runtime performance. GraphFP without the cell-cell interaction term will be efficient for large number of cell types since the degree of freedom (e.g., the parameters of linear potential energy Φ) will grow in the order of O(n). For example, the runtimes of GraphFP without cell-cell interaction term (W = 0) for both the murine cerebral cortex dataset and the mouse spinal cord injury dataset are all less than 20 seconds (Tables S2 and S3).” (Lines 433-454 on page 16 of the main text, Lines 455-466 on page 18 of the main text).

(2) In our implementation, as the existing methods (e.g., Waddington-OT [15] and Tempora [12]), the cell clustering and annotation were performed on the entire data by merging cells from all time points together. Such approach offers an unbiased, comprehensive and quantitative definition of discrete cell types. We discussed the possibility of clustering the single-cell time series scRNA-seq data in different batches in a serial fashion as follows:

“Secondly, as the existing time series scRNA-seq methods such as Waddington-OT [15] and Tempora [12], the current GraphFP works in an off-line fashion such that the cell clustering and annotation are performed on the entire data by merging cells from all time points together. This approach offers an unbiased, comprehensive and quantitative definition of discrete cell types. However, with the emerging large-scale scRNA-seq data, it may be computationally cumbersome to cluster the massive and continually arriving scRNA-seq datasets as a whole. Therefore, developing an on-line framework of GraphFP that can cluster and annotate the single-cell time series scRNA-seq data in different batches in a serial fashion should be an interesting topic. The newly developed single-cell data analysis tools such as the on-line integration method online iNMF [40] and the cell type annotation method scArches based on transfer learning [41] can be adopt.” (Lines 521-533 on page 19 of the main text)

2. Quantification of the cell stemness. This is an important issue in scRNA-seq data analysis. Will the proposed method give a quantification on this problem? I would like to see some tests and comments on this point.

Reply: Thank you for your valuable suggestion. In the revised manuscript, we addressed the quantification of the cell stemness in a newly added section “The linear potential energy Φ by GraphFP quantifies cell differentiation potency” (on pages 10-11 of the main text and Fig 3 on page 12 of the main text) as follows:

“Computational quantification of cell differentiation potency (as known as cell stemness) is a challenging issue [18]. The pioneer work by Shi et al. [5] established a rigorous mathematical theory on quantifying cell stemness from scRNA-seq data based on continuous birth-death process.

Here, we demonstrated that the linear potential Φ estimated by GraphFP can be used to quantify the cell differentiation potency. In this study, each cell will be assigned the same linear potential value as that of its corresponding cell type/state. Shi et al. [5] also quantified the cell differentiation potency at the cluster level (e.g., cell state and cell type), which makes the results more accurate and robust. Quantifying the cell differentiation potency at single-cell level is still difficult, as the single-cell gene expression profiles are known to be error-prone due to various technique issues [31]. Following the study in Shi et al. [5], we tested whether our linear potential energies for pluripotent stem cells (at early time point) are higher than those for differentiated cells (at latter time point). It is clearly shown that, cells from samples collected at earlier time stages tend to have higher potential Φ and vice versa (Fig 3(a)). When using the one-sided Wilcoxon

ranksum statistic as applied by Shi et al. [5], we confirmed with highly statistically significant results that the linear potential values of cells sampled at the earliest time stage E11.5 are higher than those cells sampled at the subsequent time stages E13.5 ($p < 1.554e-07$), E15.5 ($p < 2.2e-16$), and E17.5 ($p < 2.2e-16$), respectively.

Next, we checked the linear potential energies of the cell types with their pseudo-time during cell development process. Tempora [12] provided each cell type with a temporal score by adjusting its cell composition from each time point such that a cell type containing more cells from an early time point will have a low score and vice versa. We therefore used the temporal scores as the pseudo-time for the 7 cell types. It is clear demonstrated that the addictive inverse values of linear potential energy are strongly correlated with the temporal scores (Fig 3(b), Pearson correlation coefficient=0.91), further confirming that the linear potential energy well quantifies cell stemness. It is worth noting that the linear potential energy $\Phi$ (Fig 3(c)) is different from the stationary distribution **Pss** of cell types (Fig 3(d)). The stationary distribution **Pss**, which is the cell type frequencies or cell densities calculated using the merged data across all time points, is often used to construct the stationary energy landscape **Uss** $\equiv -\log$ **Pss** in scRNA-seq data analysis [6]. However, as pointed out by Shi et al. [5], the stationary energy landscape **Uss** is the equilibrium potential induced by diffusion without birth and death. In some extent, the linear potential energy $\Phi$ by GraphFP is an analogy of the cell potential V (x) proposed by Shi et al. [5] which was taken as their quantification of cell differentiation potency." (see Lines 261-271 on page 10 and Lines 272-299 on page 11 of the main text)

3. The authors choose the optimal transport (OT) approach and and Wasserstein distance to the scRNA-seq data analysis, which is a hot topic in this area and machine learning community. Yes, it is fancy to take OT. But it is also possible to utilize other simpler model and optimization approach to accomplish similar task. I wonder whether the authors have some reason to take OT instead of other methods. Some comments and discussion on this point should be added.

Reply: Thank you for your valuable suggestion. Beyond the theoretical importance of OT, the main reason that we developed GraphFP based on the fundamental form of OT (a nonlinear FPE on graph) is that, such framework allows GraphFP to learn the complex geometry of data, as well as to provide a novel way to quantify cell-cell interactions during cell development. In the revised manuscript, we addressed this point in both Introduction and Discussion parts as follows:

1) "An emerging number of methods are being developed to reconstruct cell developmental energy landscape from time series single-cell data using the mathematical framework of optimal transport. Optimal transport has received considerable attention in recent years for various disciplines such as machine learning and statistical data analysis, as it has been proven to be a powerful tool in the analysis of complex data [13]. The core concept of optimal transport, Wasserstein distance between two probability distributions, quantifies an optimal cost of transporting one data distribution to the other. As a remarkably rich and fruitful concept, Wasserstein distance "enables a mechanism transforming the probability space into a Riemannian manifold (known as a Wasserstein manifold), so that geometric structures and partial differential equation (PDE) techniques can be established and analyzed" [14]." (Lines 29-39 on Page 2 of the main text)

2) "Building upon the fundamental form of optimal transport, GraphFP learns the complex geometry of data, as well as provides a novel way to quantify cell-cell interactions during cell development. It models the cell developmental process as stochastic dynamics of the cell-state/-type frequencies on probability simplex in continuous time. The discrete Wasserstein distance is introduced to transform the probability simplex into a Riemannian manifold, called discrete Wasserstein manifold. The FPE is

proven to be the gradient flow of the free energy on the discrete Wasserstein manifold." (Lines 56-63 on Page 3 of the main text)

3) "In this study, we propose GraphFP, a state manifold based computational framework, to reconstruct the complex potential energy landscape and infer the stochastic dynamics of cell state-transition during cell development. GraphFP models cell development based on the diffusion process in a discrete spectrum of states [19–21]. It can be viewed from the lens of dynamic optimal transport on networks as solving an optimal control problem to minimize the kinetic energy of flow between adjacent time points [14,39]. The FPE of GraphFP can be characterized as a gradient flow of free energy when the probability simplex of discrete states is equipped with the discrete L2-Wasserstein metric defined on the graphs [19–21]. Beyond its clear theoretical importance, GraphFP has enabled critical insight into nonlinear dynamic cell state-transition, as well as cell-cell interactions during cell development. We demonstrated that the cell-cell interaction part of GraphFP plays a key role in capturing the stochastic dynamic of the cell-type frequencies on both the murine cerebral cortex dataset (Table 1, Fig 5) and the mouse spinal cord injury dataset (Supplementary Note 3 and Table S1)." (Lines 484-497 on Page 18 of the main text)

Minor:

1. Some typo or gramatical errors.
Line 73: double periods in the text.
Line 121: as follows

Reply: Thank you for pointing out these errors! We have corrected them in the revised manuscript.
