# Supplementary material for: Dynamic inference of cell developmental complex energy landscape from time series single-cell transcriptomic data
Source: PLoS Comput Biol. 2022 Jan 24;18(1):e1009821. doi: 10.1371/journal.pcbi.1009821 (PMC8812873; doi:10.1371/journal.pcbi.1009821)
Supplement: Attachment — Submitted filename: ResponseLetter-GraphFP-R2.pdf [file pcbi.1009821.s006.pdf]

Responses to Reviewers:

We would like to thank the reviewers for thoughtful comments and valuable suggestions which have greatly helped us to improve our manuscript. The manuscript has been revised by following the reviewers' comments and suggestions, making our results more solid and convincing.

The point-by-point responses to the reviewers' comments and suggestions are as follows and our responses are in red color.

Reviewer #1: The authors have addressed my concerns thoroughly. I have only a few minor comments.

Reply: Thank you very much for your comments and suggestions, which have greatly improved our paper.

1, Line 483, is ref. 37 the right reference here? It seems that ref. 37 is not talking about data-driven landscape.

Reply: Thank you very much for your comment! In the revised manuscript, we have replaced the ref. 37 on Line 484 with the ref.18 which addressed the data-driven landscape (Line 484 on page 18 of the main text).

2, The final word of the paper (Line 533), "adopt" should be adopted?

Reply: Thank you for pointing out this error. We have corrected "adopt" on Line 534 to "adopted" in the revised manuscript (Line 534 on page 19 of the main text).

3, Fig. 3 (c and d), the y axis seems to be labeled incorrectly.

Reply: Thank you for pointing this out. We have corrected the labels of y-axis for Fig. 3c and Fig. 3d in the revised manuscript (on page 12 of the main text).

Reviewer #2: The authors responded to my concerns perfectly. Now I recommend it for publication.

Reply: Thank you very much for your comments and suggestions, which have greatly improved our paper.
